# Dr. Waters

**Published:** 1894-01

**Authors:** 


**Dr. H. W. Waters,** of Brenham, the oldest resident physician of that county, and a prominent member of the Texas State Medical Association, was killed on the streets of Brenham on the 4th of January, inst., by a stray bullet. He was hastening to separate two lads who were fighting, when one of them fired a pistol, with the result stated.

